# Comparison of Physical and System Factors Impacting Hydration Sensing in Leaves Using Terahertz Time-Domain and Quantum Cascade Laser Feedback Interferometry Imaging

**DOI:** 10.3390/s23052721

**Published:** 2023-03-02

**Authors:** Khushboo Singh, Aparajita Bandyopadhyay, Karl Bertling, Yah Leng Lim, Tim Gillespie, Dragan Indjin, Lianhe Li, Edmund H. Linfield, A. Giles Davies, Paul Dean, Aleksandar D. Rakić, Amartya Sengupta

**Affiliations:** 1 Department of Physics, Indian Institute of Technology Delhi, New Delhi 110016, India; 2 DRDO-Industry-Academia Center of Excellence, Indian Institute of Technology Delhi, New Delhi 110016, India; 3 School of Information Technology & Electrical Engineering, The University of Queensland, Brisbane, QLD 4072, Australia; 4 School of Electronic and Electrical Engineering, University of Leeds, Leeds LS2 9JT, UK

**Keywords:** terahertz imaging, time-domain spectroscopy, laser feedback interferometry, agriphotonics

## Abstract

To reduce the water footprint in agriculture, the recent push toward precision irrigation management has initiated a sharp rise in photonics-based hydration sensing in plants in a non-contact, non-invasive manner. Here, this aspect of sensing was employed in the terahertz (THz) range for mapping liquid water in the plucked leaves of *Bambusa vulgaris* and *Celtis sinensis*. Two complementary techniques, broadband THz time-domain spectroscopic imaging and THz quantum cascade laser-based imaging, were utilized. The resulting hydration maps capture the spatial variations within the leaves as well as the hydration dynamics in various time scales. Although both techniques employed raster scanning to acquire the THz image, the results provide very distinct and different information. Terahertz time-domain spectroscopy provides rich spectral and phase information detailing the dehydration effects on the leaf structure, while THz quantum cascade laser-based laser feedback interferometry gives insight into the fast dynamic variation in dehydration patterns.

## 1. Introduction

**Figure 1:**
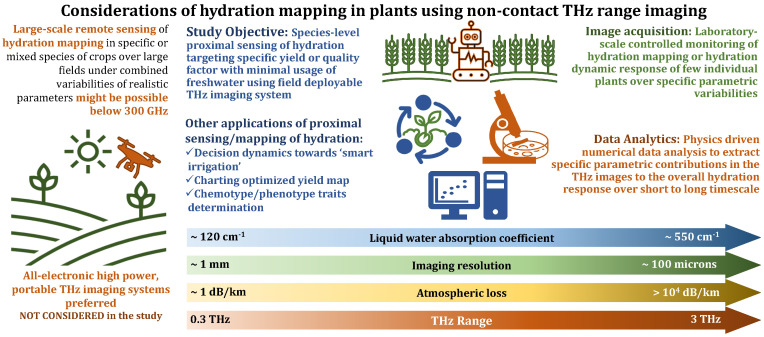
Approaches toward THz hydration mapping in plants.

The recently published annual report ‘The State of Food and Agriculture 2022’ by the Food and Agriculture Organization of the United Nations [1] highlights the acute crisis of high to very high water stress and argues for the use of sensors to monitor plants in order to support humans making decisions on agricultural tasks, including the triggering of irrigation. Because irrigation accounts for 80% of the available freshwater consumption worldwide, [2] principally in the agricultural sector, ‘smart’ irrigation management can be a potential way to mitigate the impending food and water crisis in the coming decades which is fueled by a burgeoning world population and climate change. This is further emphasized by the impact of climate change on the amount of water available for irrigation, leading to the increasing need to assess the water requirement, and to optimize its use. This will lead to what is frequently referred to as the ‘fourth agricultural revolution’, or ‘Agriculture 4.0’ [3,4]. This concept, in recent years, has led to the development of precision farming or agriculture, which aims to optimize the use of natural resources, such as water, soil, energy, etc., while maximizing the yield [5]. One of the essential elements of this dogma of precision farming is the fast, reliable, and accurate sensing of various parameters, as shown in Figure 1. The traditional methods for WC estimation in plants were guided by monitoring the soil moisture level and weather condition. This was an indirect scrutiny technique; hence, it did not provide an accurate estimate of the WC and suffered from a lack of specificity with regard to an individual plant specimen [6]. Moreover, commonly used standard direct hydration detection techniques, including the gravimetric WC method, direct drying and distillation, are destructive and time-consuming [7]. Photonic-based measurement techniques, which inherently possess all these attributes, are therefore increasingly being used both in laboratory-scale and large-scale field settings [8]. These approaches, through systematic data acquisition followed by extensive data analyses, lead to viable pathways toward realistic implementations of ‘smart’ management methods through the formation of hydration models.

In the case of liquid water sensing in plants, terahertz (THz) frequencies were already employed [9,10,11,12,13] due to the unique combination of the attributes of this spectral range: high absorptivity of liquid water; limited transparency through most dielectrics, including paper, cloth, and plastics; and low-energy, non-ionizing photons causing no harm to the users over long exposures. At the same time, the complex THz spectral response from the plant, generated due to strong scattering attenuation, water vapor absorption in free-space THz transmission, and resonant absorption by biomaterials other than water [14,15], necessitates extensive data analyses for a correct interpretation [16].

In addition to spectral information, successful hydration mapping, especially in leaves, is equally possible spatially in the true THz range with a sub-mm resolution, a resolution hard to achieve in the sub-terahertz spectral range. Due to water being the most common constituent present in various parts of the leaf, a THz response obtained in both the transmission and reflection can provide a sharp contrast. This occurs, in particular, due to the strong absorption of THz radiation by liquid water [17,18] in the vein structures, with relatively higher transparency occurring in regions of the leaf consisting mostly of cell groups, which contain a much lower concentration of liquid water (the flat component of the leaf, the lamina) [19]. Additionally, the reason for their transparency to THz waves lies in the fact that their cell walls are mostly made of cellulose which has a relatively lower absorption in the THz range [12].

Apart from these considerations, the ultimate challenge is the implementation of THz technology as a portable, rapid, and reliable approach for hydration sensing/mapping, either on the laboratory scale or in the field, where uptake remains low [20,21]. Some of the system-level constraints include the overall form factor, weight, and cost of imaging systems; the source power and detector sensitivity of the THz technologies utilized therein; as well as the associated data acquisition and analyses times [22,23,24]. Therefore, it is critical that a comparative study of contrasting THz imaging platforms, both broadband and single frequency, be undertaken in a methodical manner to determine the technical feasibility and comparative advantages of different THz imaging techniques [25,26], in terms of the acquisition and analyses. Such will engender the future toward the practical utilization of THz technologies in hydration mapping applications.

So far, THz time-domain spectroscopy (THz TDS) is the most extensively utilized technique for monitoring water content in plants with well-developed methodologies and computational tools. However, THz quantum cascade laser-based laser feedback interferometry (THz-QCL-LFI) is a quite recent addition as a THz imaging technique; thus, only a few papers have reported its application for hydration sensing in plants [25]. It would be of great significance to directly compare the two distinct techniques to understand their merits and demerits for hydration-sensing applications. Henceforth, their feasibility and viability can be assessed toward precision agriculture. However before quantitative hydration measurement can be achieved, it is essential to understand the advantages and limitations of these two techniques under study, including the configuration, acquisition speed, spatial resolution, and others. With this objective, in our present work, we investigate these two THz techniques to create a high-resolution hydration map in different species of plants ex vivo. Broadband THz imaging was employed to investigate the dehydration pattern of Indian Bamboo (*Bambusa vulgaris*). Comparatively, fast narrowband THz-QCL-LFI imaging was utilized to investigate the dynamic evolution of the dehydration patterns in Chinese celtis (*Celtis sinensis*). We have opted to use two leaves of very different structures to establish the general trends from the two measurement techniques and identify the relative advantages and disadvantages inherent to each technology and discover how they complement each other.

## 2. Leaf–THz Interaction

A plant leaf can be visualized as a small biological reactor with several physiological processes undergoing simultaneously. Any leaf is made up of mainly three components: air, water, and plant material (cellulose with a small amount of fat, proteins, and sugars) [19]. These three components respond differently when exposed to THz radiation. Water, being a polar liquid, is highly absorbing in the THz range. Moreover, previous studies on the optical response of liquid water suggests that the water molecules do not exhibit a sharp absorption signature in the THz range (up to 6 THz) [27,28]. On the other hand, dry plant material, being non-polar dielectric, appears quite transparent in the same frequency range [29].

Water being the most common constituent present in various parts of a leaf, and especially in the veins, provides high contrast in the THz image in terms of both the transmissivity or reflectivity [30]. These variations in the contrast are brought upon by the strong absorption of the THz radiation by the liquid water in the vein structures, and the relatively high transparency of the parts of the leaf consisting mostly of cell groups (mostly cellulose). These cell groups also contain water but at a much lower concentration than the vein structures [14]. This means changes in the THz contrast are most likely to be associated with changes in the water content within the leaf.

**Figure 2:**
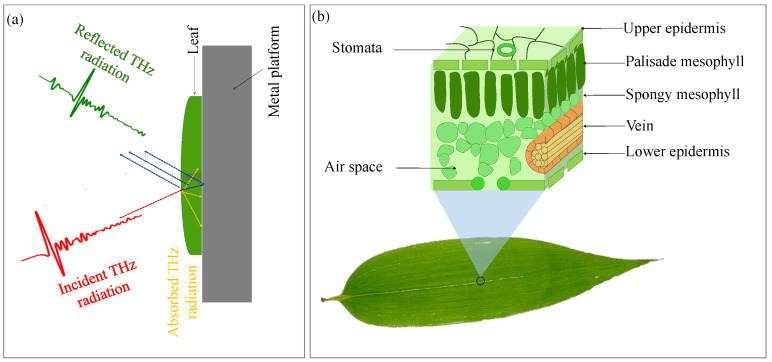
(**a**) Schematic showing interaction of THz radiation with the leaf sample (mounted on a metal platform) in reflection configuration and (**b**) the internal structure of a typical leaf and its main components.

As the schematic in Figure 2a shows, the THz radiation is incident on the upper epidermal surface of the leaf. A portion of this incident THz wave is reflected from the front surface while the rest is either reflected from the sub-surface structure or gets absorbed by the sample [31]. For severely drought-stressed leaves, part of the radiation could be transmitted through a certain portion of the leaf (away from the veins and nearer the edges) and then get reflected from the metal backing surface. The reflectivity of the leaf sample is affected by the amount of water present in it. It is well-known that depending on the nature of the tissue, the water level changes in different parts of the leaf. For instance, the epidermis and the mesophyll contain a smaller amount of water (see Figure 2b), while the veins of the leaves are responsible for the transportation of water from the root and stem to throughout the plant and hence contain a large amount of water [32]. In other words, we can say that the spatial distribution of water in a leaf is not even. In THz images, the spatial distribution of water can be seen in terms of the image contrast.

Thus, the response of the leaves recorded in the THz spectroscopic or imaging setup represents complex, convoluted data which, oftentimes, strongly depends on outside conditions, such as the temperature, humidity, pressure, and pH of the surrounding media. One must remember, however, that THz imaging techniques in general, including the ones which are presented here, are not ‘single-shot capture’ methods. There is a finite time of acquisition which may vary from seconds to hours, as in one of our cases. Consequently, in the process of the acquisition of the leaf’s image, the natural hydration dynamics (whether of the drought-stressed plant or unstressed plant or of the plucked leaf) continues and the exact amount of hydration in the leaf continues to vary within that time frame.

## 3. Experimental Setup

### 3.1. Broadband THz TDS Imaging

**Figure 3:**
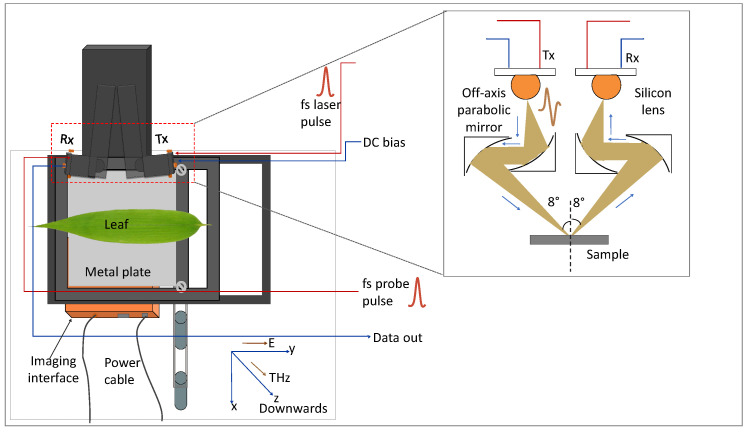
Schematic of the THz TDS imaging setup with leaf samples mounted on the metal platform in reflection configuration (the schematic in the inset is the zoomed-out area shown by the red box. It shows the propagation of THz radiation from the Tx to the sample and then from sample to the Rx).

Figure 3 shows the broadband THz TDS imaging setup (Teraflash Pro, TOPTICA Photonics AG.) utilized in this work. The THz generation and detection schemes are based on femtosecond (fs) laser-driven photoconductive antenna (PCA). Peak power of the driving fs laser pulse is 80 mW with a repetition rate of 100 MHz. The laser pulse splits into two with the help of a beam splitter; a part of it is directed toward the emitter PCA and the other part was sent to the detector. THz radiation is generated at the emitter PCA by acceleration of photocarriers (generated by the fs optical pulse) by means of an external DC bias (100 V) applied at the antenna [33].

Average power of the generated THz pulse was 60 µW with a pulse width of 0.6 ps. The THz signal was directed and focused on the sample with the help of a pair of off-axis parabolic mirrors (OAPMs) and another pair of OAPMs was utilized to guide the THz radiation from sample to the detector. At detector PCA, the THz signal was sampled by the other part of the optical pulse. The peak dynamic range of the approaches 90 dB at 0.9 THz. The angle of incidence of the THz beam in reflection mode was 8°.

**Figure 4:**
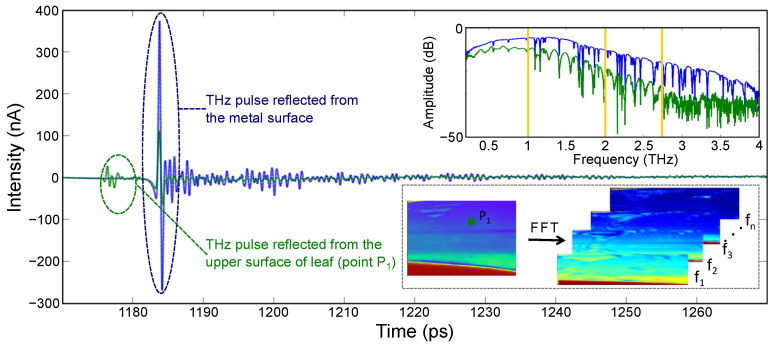
Schematic showing the post-processing of experimental data: typical experimentally obtained time-domain signal and images in broadband THz TDS imaging setup and the post-processed amplitude and phase images.

To obtain the amplitude and phase information of the signal, the experimental images were converted to frequency domain (by taking FFT) using Matlab. In PCA-based THz-TDI system, a broadband spectrum was recorded over the bandwidth of the system as shown in Figure 4. With the intention to monitor the leaf hydration level at different frequency instances, the amplitude and phase images were obtained at different frequency points, namely 1.0, 2.0, and 2.75 THz. The frequency lines for which the images were extracted are shown with orange lines in the frequency spectrum. Afterward, the amplitude and phase images were normalized with respect to the maximum value obtained from the image.

### 3.2. Fast THz LFI Imaging

**Figure 5:**
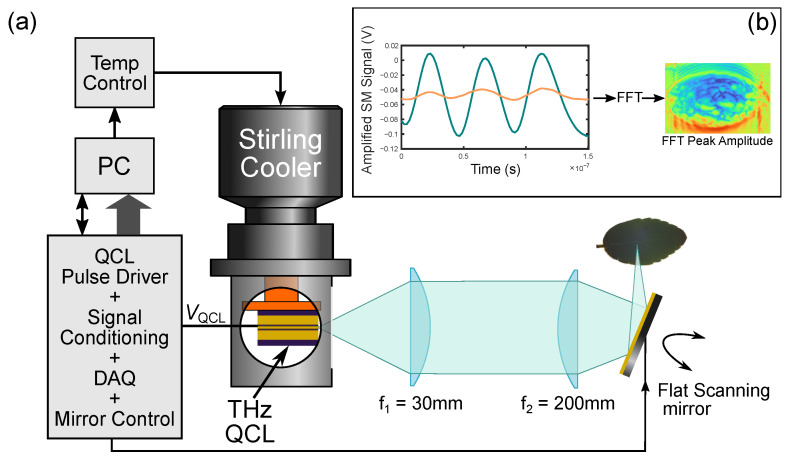
(**a**) Schematic of THz QCL LFI imaging setup showing fast scanning imaging mode. (**b**) Processing steps of the LFI interferogram (amplified self-mixing (SM) signal) into image pixels. Blue trace shows signal off bare metal backing. Orange trace shows representative signal from the leaf.

Laser feedback interferometry is a versatile technique which allows a laser—such as a THz QCL—to be used as both a source and detector of emitted radiation [24]. The nature of the LFI scheme takes advantage of the coherent property of the THz QCL, suppresses background radiation (which can be a problem for many external THz detectors [23]), and delivers a compact, self-aligned, highly sensitive interferometric THz transceiver [34,35]. When applied to imaging with appropriate calibration, the LFI sensor can be used to extract complex optical properties of the target [36,37,38,39]. Figure 5a shows the schematic of the QCL-based LFI imaging setup. The operating frequency of the THz QCL was 2.71 THz (measured via the method shown in [40]). A custom-built pulsed laser driver was used to drive the QCL. A mechanical Stirling engine cryocooler (Cryotel GT, Sunpower, Inc., Athens, USA) was used to maintain the operating point of QCL at 50 K. The THz radiation generated by the QCL was collimated (L1 Tsurupica *d* = 25 mm *f* = 50 mm) and focused (L2 TPX *d* = 100 mm *f* = 200 mm) on the target, with a path length between the QCL and the sample (leaf) being approximately ∼1.2 m. The QCL was driven by a train of current pulses, each consisting of a square pulse (of 500 ns duration) on which a smaller current ramp was superimposed. During the pulse, the total laser current was changed from 1.4 A to 1.1 A, with 25% duty cycle. This current ramp (superimposed on the square pulse) induces a QCL emission frequency sweep ( 600 MHz), causing the formation of interferometric fringes that contain information about the target [41]. These fringes are extracted from the change in terminal voltage across the QCL (the self-mixing (SM) signal) [42]. Specific details of the exact driving and signal recovery process can be seen in [43]. The post-objective scanning configuration was chosen to perform the image acquisition. In this situation, a 3-inch-diameter fast scanning mirror was located between the focusing lens and the target, at 50 mm distance from the focusing lens. This configuration, while simple to implement from an optical viewpoint, has the disadvantage that the focal plane is curved and is less common than the pre-objective scanning arrangement [44,45]. However, it offers a relatively high resolution over large scan area. In this case, the scan area with a good image quality was increased from ∼10×10 mm to ∼25×25 mm (while using the same *f* = 200 mm lens) by replacing the pre-objective with the post-objective scanning architecture. For an interferometric imaging system like the LFI, the filed curvature manifests itself through the appearance of dominant concentric equiphase areas, rendering the useful phase information more difficult to interpret. Without the additional correction technique, the fast scanning LFI essentially becomes a predominantly amplitude detecting scheme.

For the QCL LFI images, the interferogram for each pixel is filtered and the amplitude recovered from the dominant peak of the FFT (see Figure 5b). The limited laser frequency shift created by the current sweep means these values correspond to the amplitude, essentially, at the laser operating frequency of 2.71 THz.

## 4. Results

### 4.1. Broadband TDS Imaging Results

The leaf samples for the THz TDS imaging were collected from the Indian bamboo plant cultivated in the subtropical region, with geographical coordinates 28.550 N Lat., 77.193 Long. (Indian Institute of Technology Delhi, India). During the sample collection, the temperature and humidity recorded were 30 °C and 55%, respectively. The images were acquired in the laboratory where the humidity was maintained between 30–40% and the temperature was maintained at 22 °C. The first set of images of the leaves were acquired 30 min after the leaves were plucked. Afterward, two more sets of images were acquired, starting 180 min and 360 min from plucking. It took around 75 min for each image acquisition.

The quantity and quality of the information incorporated in the frequency-dependent amplitude and phase images depend on the penetration depth, resolution, and signal-to-noise ratio of the THz radiation at the frequencies of interest. It is a well-known fact that as one moves forward on the frequency axis, the penetration depth of the THz radiation decreases while its resolution increases. Due to the variation in the penetration depth with the frequency, one can visualize the frequency-dependent THz TDS images as pseudo-tomographic images that show information at different depths of the leaf. The term ‘pseudo-tomographic’ is used because we are not considering the true reflectivity of the leaf here. Instead, the THz response at each pixel is the convoluted effect of a reflected and double-transmitted signal. Moreover, at higher frequencies, the THz power decreases which results in a decrease in the signal-to-noise ratio (SNR). Thus, the THz TDS images at higher frequencies lack sensitivity toward a change in the water content, and the information is limited to only the surface or sub-surface regions of the leaf.

**Figure 6:**
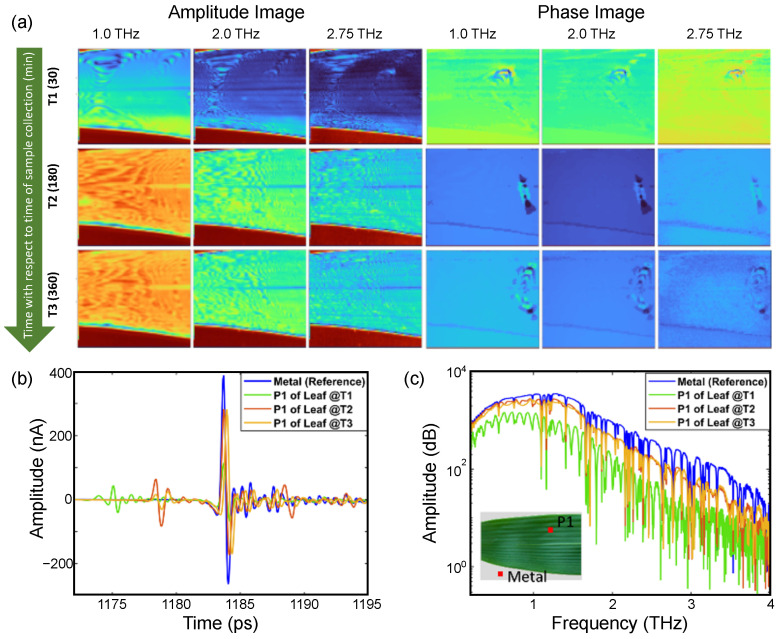
(**a**) Amplitude and phase images of a Bambusa vulgaris leaf acquired after 30 min (T1), 180 min (T2), and 360 min (T3) from plucking, extracted at different frequency points. The color contrast in amplitude image represents variation in reflectivity while the color contrast in phase image represents the variation in phase accumulation. For a continuous progression from 1 to 3 THz, see Video S1, and (**b**) THz time-domain response and (**c**) amplitude spectrum of leaf (from position P1) and metal acquired at time T1, T2, and T3, showing degree of dryness in the leaf. The visual image of the leaf is shown in the inset of the amplitude spectrum and the data were extracted from pixels shown in red in the inset.

Figure 6 shows the amplitude and phase images of the bamboo leaf at three different frequency lines (shown with red lines in the frequency spectrum of Figure 4a). The bare metal, being close to an ideal reflector of THz radiation, appears in the red color in the amplitude images. As the images were raster scanned, the image acquisition time was long enough for the leaf to go through a significant change in the water content. This is observed as a light color gradient leaf image while moving from top to bottom (particularly prominent in the amplitude images at T1). Video S1 shows the 180 min time period but sweeps continuously through the 1 to 3 THz (50 GHz step) capture by the TDS system.

Unlike the amplitude images, for the phase images the contrast increases with frequency (where the phase presented in the images is the phase accumulation at each pixel) (see Video S1). This increase in the contrasts is due to the two major components of the plant material, one being water and the other being cellulose. The nature of cellulose causes an increase in the phase accumulation with increasing frequency, while at the same time the absorption of water also increases, leading to a decrease in the accumulated phase. These competing features lead to the higher contrast observed in the phase images. For a better understanding, the THz time-domain waveform (left panel) and the amplitude spectrum are shown in Figure 6c. As shown in the TD waveform, the amplitude of the signal reflected from the metal surface (double transmitted through the leaf) increases as the leaf dehydrates because of the reduced absorption by the leaf. The amplitude spectrum depicts the same response, and the reflected amplitude increases with the loss of water in the leaf from T1 to T2. There is no significant difference in the THz response between T2 and T3, which is also illustrated by the THz images. The anomaly observed in the phase images is due to a crease in the leaf and changes with time as the leaf dehydrates, crinkles, and curls.

**Figure 7:**
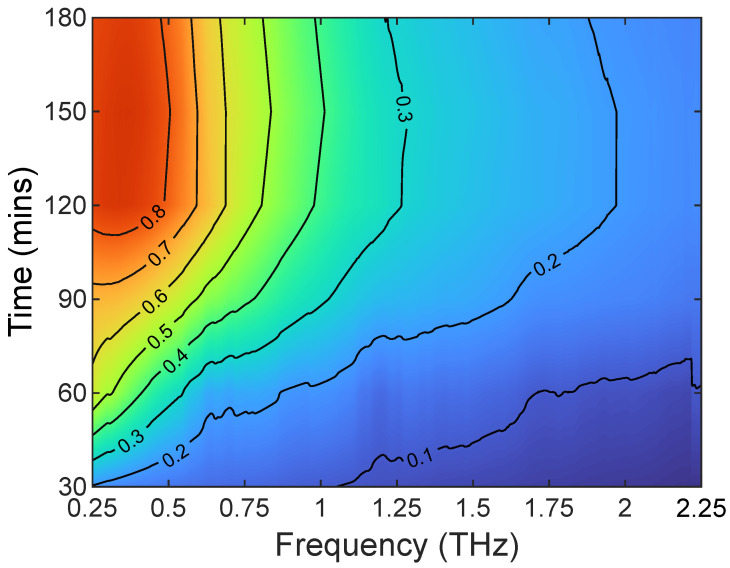
Time vs. frequency plot of calculated penetration depth for a *Bambusa vulgaris* leaf. Contours are penetration depth in mm.

A particular advantage of TDS is not only being able to determine where from in the leaf the signal is coming but to also work out from the absorption coefficient what hydration level and thickness of the leaf leads to what depth with frequency. Figure 7 shows this change in the calculated penetration depth vs. frequency and time for a single measurement. As the leaf is drying out over time, the change in time is really representative of a change in hydration. This is important as it can then be used inversely to estimate the thickness of the leaf and relate what is the frequency of the leaf spectral reflectivity, transmissivity, or a combination of both.

### 4.2. Fast LFI Imaging Results

**Figure 8:**
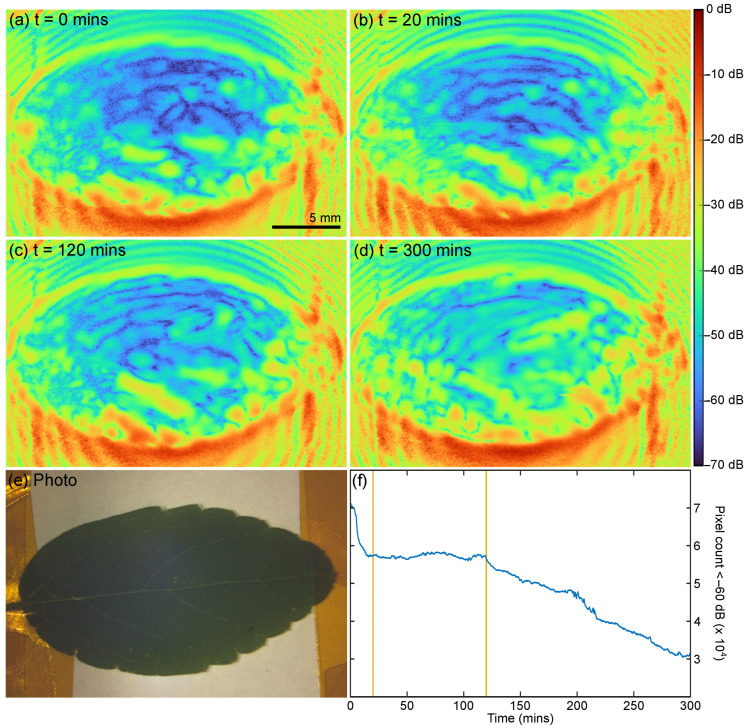
(**a**–**d**) LFI images of *Celtis sinensis* at t = 0, 20, 130, and 300 min. For a continuous progression from 0 to 300 min, see Video S2. (**e**) Photo of leaf after LFI scan (300 min +). (**f**) Change in count of ‘blue’ pixels (Amp. < 60 dB) vs. time. Horizontal lines indicate 20 and 120 min.

For the QCL-LFI study, leaves were collected from a Chinese celtis seedling, from −27.500 S Lat., 153.0153 Long. (The University of Queensland, St Lucia, Australia). The leaves were collected and mounted, and the imaging started in under 2 min after plucking. As with the TDS measurements, the leaf was attached to a metal substrate. Each image acquisition required approximately 25 s and was continuously repeated for about 5 h. Figure 8a–d show the resulting images at certain time points (0, 20, 120, and 300 min) and Figure 8e shows a photo of the leaf after the scans were completed (five hours after collection). Video S2 is an animation containing the images acquired over the initial 300 min—the entire scan in one frame per 25 s. In Figure 8a–d, the blue pixels are associated with low reflectivity areas (with a reflectivity amplitude of 60 dB and more below the peak reflectivity), suggestive of high water content and high absorption. Adding all these pixels defines the fraction of the leaf with high water content. Therefore, observing the temporal evolution of the count shows the change in the total water content in the leaf over the imaging time, see Figure 8f.

Our TDS measurements show (see Figure 4) that the total reflected THz signal is dominated by the reflection from the metal substrate combined with the double transmission through the leaf. Therefore, one can clearly associate the low reflectivity with higher absorption in the leaf and consequently a lower total reflectivity of the leaf-substrate assembly. Based on this, we have constructed the hydration map, depicting the change in the water content in the leaf with time (Figure 8a–d). Moreover, this allows for insight into the process of water loss in the plucked leaf with time, including the ‘effort’ the leaf undertakes to conserve water when under stress. What is obvious from the images, Video S2, and the blue pixel count is the leaf undergoes several different stages as it dehydrates.

If we assume that the low reflectivity of the leaf-substrate assembly is an indicator of higher hydration, we can map the change in the water content in the leaf with time and potentially observe the process the leaf undertakes to conserve water when under stress. What is obvious from the images, Video S2, and the blue pixel count is the leaf undergoes several different stages as it dehydrates.

A rapid change is observed in the first 20 min—the leaf is acting like it is still connected to the plant, presumably transpiring and photosynthesizing; then the leaf stabilizes with only a marginal loss of water content; the leaf closes the stomata and tries to stabilize the water content, perhaps in response to the lack of new water/nutrition due to being separated from the plant; and then finally, at about 120 min, it resumes to a continuous slowly decay which continued until the scanning was finished at about 300 min and presumably beyond. It is important to note that during all these stages, the reflectivity across the leaf was always changing. If one was to only observe one small area of the leaf, the larger trends would not be properly observed; similarly, if the scan time (currently 25 s) was only an order of magnitude longer, these effects would also be less pronounced or visible.

The changes in reflectivity can also be mapped to the structure of the leaf; Video S2 has a transparent mask from the photo of the leaf superimposed on it, allowing the leaf veins to be observed at the same time as the THz reflectivity. As can be seen (in Video S2), the low-reflectivity regions (areas of high water content) move along the leaf veins and inside the cells bound by the veins—similarly, certain cells become highly reflective (areas of low water content) while neighboring cells are not.

## 5. Conclusions

In this article, we used two very different THz imaging platforms for the qualitative estimation of the hydration level in plucked leaves. One is a well-established, commercially available technology, the TDS spectroscopy, and another is still being developed, the THz-QCL-LFI. We believe that we have demonstrated the relative advantages of these two platforms, summarized in (see Table 1): while the TDS system allows for depth sectioning due to its time gating capacity, it is intrinsically limited to slow image acquisition times (close to 75 min per image in this case). This clearly prohibits longitudinal studies, where the temporal evolution of the image is needed. The THz LFI imaging enables a full image acquisition in the matter of seconds (25 s in this case). When changes in the optical properties across the target are small during this time period, the LFI makes it possible to effectively monitor the spatio-temporal changes in the target over long periods of time, with a relatively high temporal resolution as well as spatial resolution compared to the THz TDS imaging. This enabled the tracking and visualization of the redistribution and loss of water in this case. Another critical point to observe is that at around 2.75 THz, the dynamic range of the THz-QCL-LFI is significantly higher than the THz TDS system (refer to Table 1); however, the absorption coefficient, which is another critical parameter affecting the efficacy of the system for the qualitative assessment of the hydration level, is the same. Owing to the combined effect of a high dynamic range and high absorption coefficient, the THz-QCL-LFI can predict the degree of dryness more accurately. However, in the case of a well-hydrated leaf, the applicability of the THz-QCL-LFI is limited by the lower penetration depth (high operating frequency). Therefore, in leaves with a higher value of initial water content, the THz-QCL-LFI cannot provide volume information which can be obtained at the lower frequencies of the THz TDS imaging.

The vast quantity of data generated by both techniques makes the interpretation of the results beyond general terms quite difficult. This presents a strong case for the application of machine learning techniques to investigate trends in hydration without the development of complex numerical models with limited agreement with the experiment, as suggested in recent publications [16,46,47]. Finally, the practicality of the THz-QCL-LFI would be improved by using newer feedback imaging techniques, with the potential for a room temperature operation of THz QCLs [48,49]. Clearly, both methods have their advantages and there is a benefit in combining them to yield broadband, depth-resolved images in addition to the high-frame-rate visualization and quantification of spatio-temporal changes in the water content.

## Figures and Tables

**Table 1:** Various parameters of the imaging systems used for hydration mapping.

System Parameters	THz-TDS	Fast THz-QCL-LFI
Frequency (THz)	**0.2 to 4**	2.71
	with 600 fs pulse width	with 600 MHz freq. sweep
Pixel Acq. Time (ms/pixel)	630	**0.1**
Image Size (pixels)	80×90	**500 × 500**
Image Size (mm${}^{2}$)	16×18	**25 × 25**
Image Acq. Time (s)	4500	**25**
Image Pixel Size (µm)	200	**50**
Resolvable	1.0 THz—1464 µm	
Feature	2.0 THz—732 µm	2.71 THz—**396 µm**
Resolution	2.75 THz—536 µm	
	1.0 THz—69.5 dB	
Dynamic Range	2.0 THz—60 dB	2.71 THz—**80 dB**
	2.75 THz—49.5 dB	
Recovered Information	**Amplitude and Phase**	Amplitude
Source Power (mW)	0.03 (average power)	**10 (peak)**
α of Water (cm${}^{-1}$) [18]	**120–740**	500
Tx/Rx Operation		
Temperature (K)	**300**	50

## Data Availability

Data underlying the results presented in this paper are not publicly available at this time but may be obtained from the authors upon reasonable request.

## Supplementary Materials

The following supporting information can be downloaded at: https://www.mdpi.com/article/10.3390/s23052721/s1, Video S1: THz TDS images of *Bambusa vulgaris* (at t = 180 min) showing the changes in frequency from 1 to 3 THz; Video S2: THz LFI images of *Celtis sinensis* in continuous progression from 0 to 300 min.
